# The optimal cut‐off value of five‐time chair stand test for assessing sarcopenia among Chinese community‐dwelling older adults

**DOI:** 10.1002/jcsm.13441

**Published:** 2024-02-11

**Authors:** Yu‐Hua Li, Xiu‐Hua Wang, Shi Ya, Huang Jiaoling, Nan Hua

**Affiliations:** ^1^ Xiangya Nursing School The Central South University Changsha Hunan China; ^2^ School of Health Sciences, Faculty of Biology, Medicine and Health The University of Manchester Manchester UK; ^3^ School of Nursing and School of Public Health Yangzhou University Yangzhou Jiangsu China

**Keywords:** 5CST, older adults, sarcopenia, the optimal cut‐off value

## Abstract

**Background:**

The five‐time chair stand test (5CST) as an indicator of muscle strength and physical function is the first step in assessing sarcopenia. We aimed to determine the optimal cut‐off value of the 5CST for assessing older adults with sarcopenia in the Chinese community.

**Methods:**

We used a stratified cluster random sampling method to recruit older adults from Chinese communities. The handgrip strength was assessed using an electronic handgrip dynamometer. The 5CST and gait speed were assessed by the trained researchers. The bioimpedance analysis device was used to evaluate the skeletal muscle index. We used the Asian Working Group for Sarcopenia diagnosis criteria as the gold standard. According to the receiver operating characteristic curve, we determine the optimal cut‐off value using the Youden index.

**Results:**

A total of 1027 participants were included in this analysis, including 337 men and 690 women with an average age of 70.35 ± 7.24 years. The prevalence of sarcopenia in total participants was 24.9%. The optimal cut‐off value of 5CST in the total population was 10.9 s. Stratified by age and gender, for the older adults aged 60–69 years, the optimal cut‐off values were 9.2 s in men and 10.8 s in women; for the older adults aged 70–79 years, cut‐off values were 10.2 s in men and 10.9 s in women; and for the older adults over 80 years, cut‐off values were 14.0 s in men and 11.5 s in women (all *P* < 0.001). The areas under the curve of 5CST were 0.632 in men and 0.650 in women (both *P* < 0.001). Using the newly defined cut‐off values, the prevalence of sarcopenia increased significantly (*P* < 0.001).

**Conclusions:**

We determined the optimal cut‐off value of the 5CST for assessing older adults with sarcopenia in the Chinese community, and this cut‐off can significantly improve the detection rate of sarcopenia. The cut‐off determined in our study will help community workers detect more people with sarcopenia and benefit from early intervention and management of sarcopenia in practice.

## Introduction

Sarcopenia is defined as an age‐related loss of skeletal muscle that has grave physiological and clinical consequences.^1^, ^2^ According to the report, the prevalence of sarcopenia in Chinese community‐dwelling older adults is 23.2%, and among those older adults over 80 years is as high as 40.6%.^3^ In terms of longer term clinical outcomes, sarcopenia was significantly associated with increased risks of physical limitations, falls and fractures, disability and death.^4^, ^5^, ^6^, ^7^ Patients with sarcopenia are generally unaware of their sarcopenic state until the gradual decline in muscle function becomes severe enough to be pathological, resulting in physical and functional dependence. It is important to improve the early assessment of sarcopenia.^8^

The Asian Working Group for Sarcopenia (AWGS) 2019 consensus contends that diagnosing sarcopenia requires measurements of both muscle quality and quantity. The sarcopenia algorithm in AWGS 2019 uses the five‐time chair stand test (5CST) as the preliminary evaluation indicator for the diagnosis of sarcopenia because it can reflect the muscle quality of older adults.^1^ The 5CST refers to the time required for subjects to complete five consecutive times from sitting in a chair to standing, which has been proven to be related to lower limb muscle strength, body performance, body balance and flexibility.^9^, ^10^, ^11^, ^12^ Studies suggested that poor performance on the 5CST predicted poorer physical performance and a higher risk of falling.^13^, ^14^ AWGS 2019 recommends ≥12 s as the cut‐off for low physical performance. This cut‐off is based on a review of evidence from Asian countries. Nevertheless, muscle health status can be influenced by ethnicity and lifestyle.^15^, ^16^, ^17^ Some studies have shown that the cut‐off values of 5CST recommended by the AWGS are not appropriate for the local population, and the cut‐off values of 5CST could significantly affect the detection rate of sarcopenia.^18^, ^19^ It is necessary to determine an optimal cut‐off value according to the local population. To our knowledge, at present, as there have been no reports on the optimal cut‐off point for evaluating sarcopenia with 5CST in China, whether the optimal cut‐off value recommended by the AWGS is applicable to the assessment of sarcopenia in older adults in Chinese communities remains to be studied.

We aim to determine the optimal cut‐off value of 5CST for assessing sarcopenia among Chinese community‐dwelling older adults, and we will compare the change in prevalence of sarcopenia after adjusting the cut‐off value.

## Methods

### Participants

The study was conducted in communities in Hunan Province, China, from June to September 2019. A stratified cluster random sampling method was used to recruit participants. First, Hunan Province is divided into five geographical regions: East Hunan, West Hunan, South Hunan, North Hunan and Central Hunan. East Hunan includes Changsha, Zhuzhou and Xiangtan; West Hunan includes Xiangxi Tujia and Miao Autonomous Region, Huaihua and Zhangjiajie; South Hunan includes Hengyang, Chenzhou and Yongzhou; North Hunan includes Changde and Yueyang; and Central Hunan includes Loudi, Shaoyang and Yiyang. Second, a city is randomly selected from each geographical region. Finally, two communities were selected from each city and investigated all older adults meeting inclusion criteria in each community. The inclusion criteria were as follows: (1) age ≥ 60 years; (2) be able to understand and cooperate in completing questionnaires and muscle assessments; and (3) under informed consent, voluntarily participate in and sign informed consent. The following conditions were excluded: (1) body oedema (that can affect the measurement of skeletal muscle index [SMI])^20^; (2) taking medications that affect muscle distribution (e.g., dexamethasone and glucocorticoids); and (3) wearing metal or electronic devices that affect the SMI measures. All participants voluntarily participated in the study and signed an informed consent form after being fully informed of the study content and relevant precautions. The Research Ethics Committee has approved this study (No. E201930).

### Measurements

The researchers collected general information about the patients through self‐designed questionnaires, such as gender, age and education level. Body weight was measured using a digital floor scale (JW13, Xiangshan Inc., Guangdong, China). Body height was measured using an infrared stadiometer (HT‐01, Xiangshan Inc.). Body weight was measured to the nearest 0.01 kg, and height was measured to the nearest 0.01 cm. Body mass index (BMI) was calculated by dividing body weight (kilograms) by body height squared (square metres).^21^

The handgrip strength (HS) was assessed using an electronic handgrip dynamometer (EH101; Xiangshan Inc.). Participants were asked to take a sitting position with their feet on the floor. The upper arm was flat with the chest, the forearm was in a neutral position and the elbow was bent at 90°. The participant's dominant hand held the handle of the electronic grip dynamometer with the maximum force and repeated it twice, recording the higher value for analyses.

The 5CST and gait speed (GS) were assessed by the trained researchers. We instructed the participants to sit in a chair with their legs bent at 90° and their hands crossed in front of them and stand from a sitting position without assistance. After confirming that the participants were able to change their position independently, they were asked to repeat the test five times as quickly as possible. The researchers recorded the time participants completed the 5CST. The researchers marked 10‐m straight lines on flat ground and instructed the older adults to walk in a straight line at their usual GS. The researchers recorded the time each participant walked from the 5th to the 10th metre, and the 6‐m GS is equal to 6 m divided by the recorded time.

The bioimpedance analysis (BIA) device (Inbody S10; Biospace, Seoul, Korea) was used to estimate SMI. First, the trained researchers guided the older adults to lay on a bed and told them to remove metal jewellery, such as rings, earrings and necklaces, and mobile phones. Second, electrodes were attached to the participants' wrists and ankles, and the older adults were told to remain still. After 1 min, the bioimpedance analyser can show the SMI of the limbs of older adults.

### Sarcopenia definition

We used the AWGS 2019 criteria to diagnose sarcopenia. Older adults with low muscle mass and low muscle strength and/or low physical performance were defined as having sarcopenia.^1^ The AWGS sets the thresholds of muscle strength, muscle mass and physical performance for Asians as follows: (1) low muscle strength: HS of <18 kg in females and <28 kg in males or taking >12 s to perform a 5CST for both men and women; (2) low muscle mass: SMI of <7.0 kg/m^2^ in males and <5.7 kg/m^2^ in females; and (3) low physical performance: GS < 1.0 m/s.

### Statistical analysis

All data were analysed by IBM SPSS 25.0, and a *P* value < 0.05 indicated statistical significance. Continuous variables were represented by mean values with standard deviation (M ± SD). Categorical variables were expressed by numbers and percentages, and the *χ* ^2^ test was used for statistical analysis. According to the sensitivity, specificity and Youden index of each point of the receiver operating characteristic curve, we determine the optimal cut‐off value for the screening of sarcopenia in the Chinese community. The Kappa value was used to indicate the consistency between 5CST and the gold standard.

## Results

### Characteristics of participants

**Figure 1:**
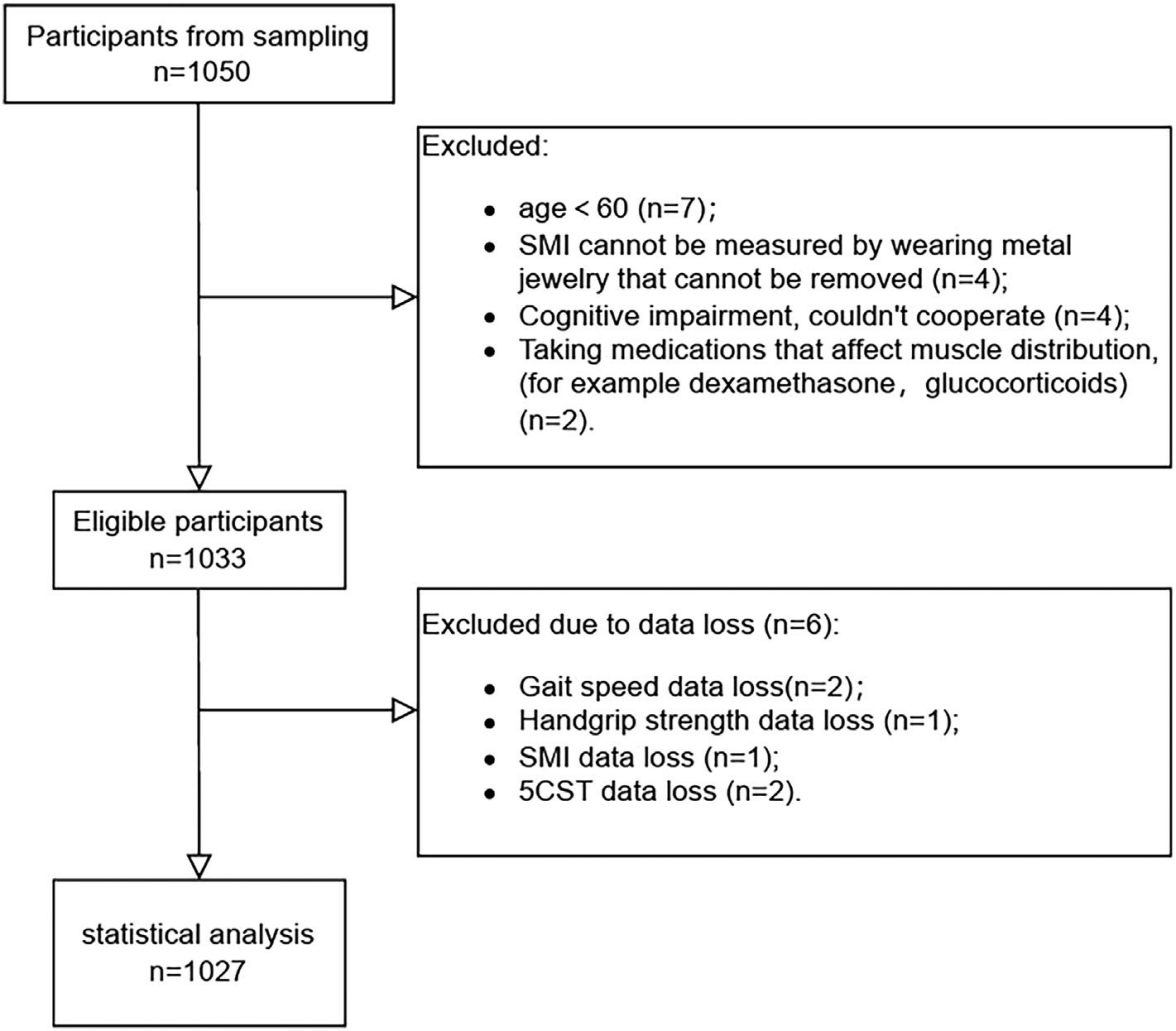
Participants' flow chart. 5CST, five‐time chair stand test; SMI, skeletal muscle index.

As shown in *Figure* *1*, a total of 1027 participants were included in this analysis, including 337 men (71.24 ± 7.10 years) and 690 women (69.92 ± 7.28 years). The prevalence of sarcopenia in total participants was 24.9%. There was no difference in the prevalence of sarcopenia between men (24.3%) and women (25.2%; *P* = 0.758). The average age of participants with sarcopenia was 74.6 years, which was significantly older than those without sarcopenia (68.95 years). Indicators related to sarcopenia, including HS, 5CST, GS and SMI, in the sarcopenia group were significantly poorer than those in the non‐sarcopenia group (*P* < 0.001 for all) (*Table* *1*).

**Table 1:** Characteristics of participants (*N* = 1027)

Variables	Non‐sarcopenia	Sarcopenia	Statistics *χ* ^2^/*t*	*P* value
Gender				
Men	255	82	0.095	0.758
Women	516	174		
Age (years)				
Men	70.01 ± 6.40	75.05 ± 7.83	5.846	<0.001
Women	68.41 ± 6.44	74.39 ± 7.79	9.109	<0.001
Height (cm)				
Men	164.22 ± 8.44	160.42 ± 6.10	3.768	<0.001
Women	153.79 ± 5.88	148.86 ± 6.03	9.436	<0.001
Weight (kg)				
Men	66.67 ± 10.70	55.17 ± 6.74	9.167	<0.001
Women	58.42 ± 7.73	46.80 ± 6.36	17.885	<0.001
BMI (kg/m^2^)				
Men	25.12 ± 8.89	21.50 ± 2.98	3.612	<0.001
Women	25.00 ± 6.95	21.12 ± 2.73	7.168	<0.001
HS (kg)				
Men	29.97 ± 8.39	21.67 ± 6.82	8.131	<0.001
Women	19.04 ± 6.05	13.85 ± 5.47	10.028	<0.001
5CST				
Men	10.08 ± 3.25	12.39 ± 5.62	4.614	0.001
Women	10.65 ± 4.07	12.51 ± 4.53	5.063	<0.001
GS (m/s)				
Men	1.03 ± 0.24	0.88 ± 0.27	4.642	<0.001
Women	1.01 ± 0.27	0.82 ± 0.23	8.364	<0.001
SMI (kg/m^2^)				
Men	7.65 ± 1.24	6.36 ± 0.49	9.148	<0.001
Women	6.39 ± 1.16	5.11 ± 0.46	20.771	<0.001

Abbreviations: 5CST, five‐time chair stand test; BMI, body mass index; GS, gait speed; HS, handgrip strength; SMI, skeletal muscle index.

### Optimal cut‐off values of five‐time chair stand test in different age groups and genders

According to *Table* *2*, the 5CST did not differ between men and women in the total population (*P* = 0.086). Stratified by age and gender, the difference in 5CST between different age groups and genders was statistically significant (*P* < 0.001). In the 60–69 and 70–79 age groups, the difference in 5CST between men and women was statistically significant (*P* < 0.05). In the age group over 80 years, the difference in 5CST between men and women was not statistically significant (*P* = 0.877).

**Table 2:** The five‐time chair stand test in different age groups and genders

Age	Men	Women	*P*_b_*
60–69	9.30 ± 2.21	9.80 ± 3.33	0.045
70–79	10.59 ± 3.79	11.81 ± 4.06	0.004
≥80	15.04 ± 6.01	14.87 ± 5.66	0.877
Overall	10.64 ± 4.07	11.12 ± 4.26	0.086
*P*_a_*	0.000	0.000	

*Note*: *P*_a_* is the statistical difference of five‐time chair stand test in different age groups. *P*_b_* is the statistical difference of five‐time chair stand test in genders.

*Table* *3* shows the optimal cut‐off values of 5CST in different age groups and genders according to AWGS 2019 as the gold standard. The optimal cut‐off value of 5CST in assessing sarcopenia in the total population was 10.9 s. Stratified by age and gender, for the older adults aged 60–69 years, the optimal cut‐off values of 5CST for the assessment of sarcopenia were 9.2 s in men and 10.8 s in women; for the older adults aged 70–79 years, cut‐off values were 10.2 s in men and 10.9 s in women; and for the older adults over 80 years, cut‐off values were 14.0 s in men and 11.5 s in women (all *P* < 0.001).

**Table 3:** Optimal cut‐off values of five‐time chair stand test in different age groups and genders

Age	Men			Women		
	Cut‐off (s)	Sensitivity (%)	Specificity (%)	Cut‐off (s)	Sensitivity (%)	Specificity (%)
60–69	9.2	73.7	51.5	10.8	48.1	74.4
70–79	10.2	57.5	58.4	10.9	62.7	51.5
≥80	14.0	65.3	66.7	11.5	80.0	35.1
Overall	9.2	76.8	44.3	10.9	62.6	63.8

### Changes in the prevalence of sarcopenia using optimal cut‐off values

**Figure 2:**
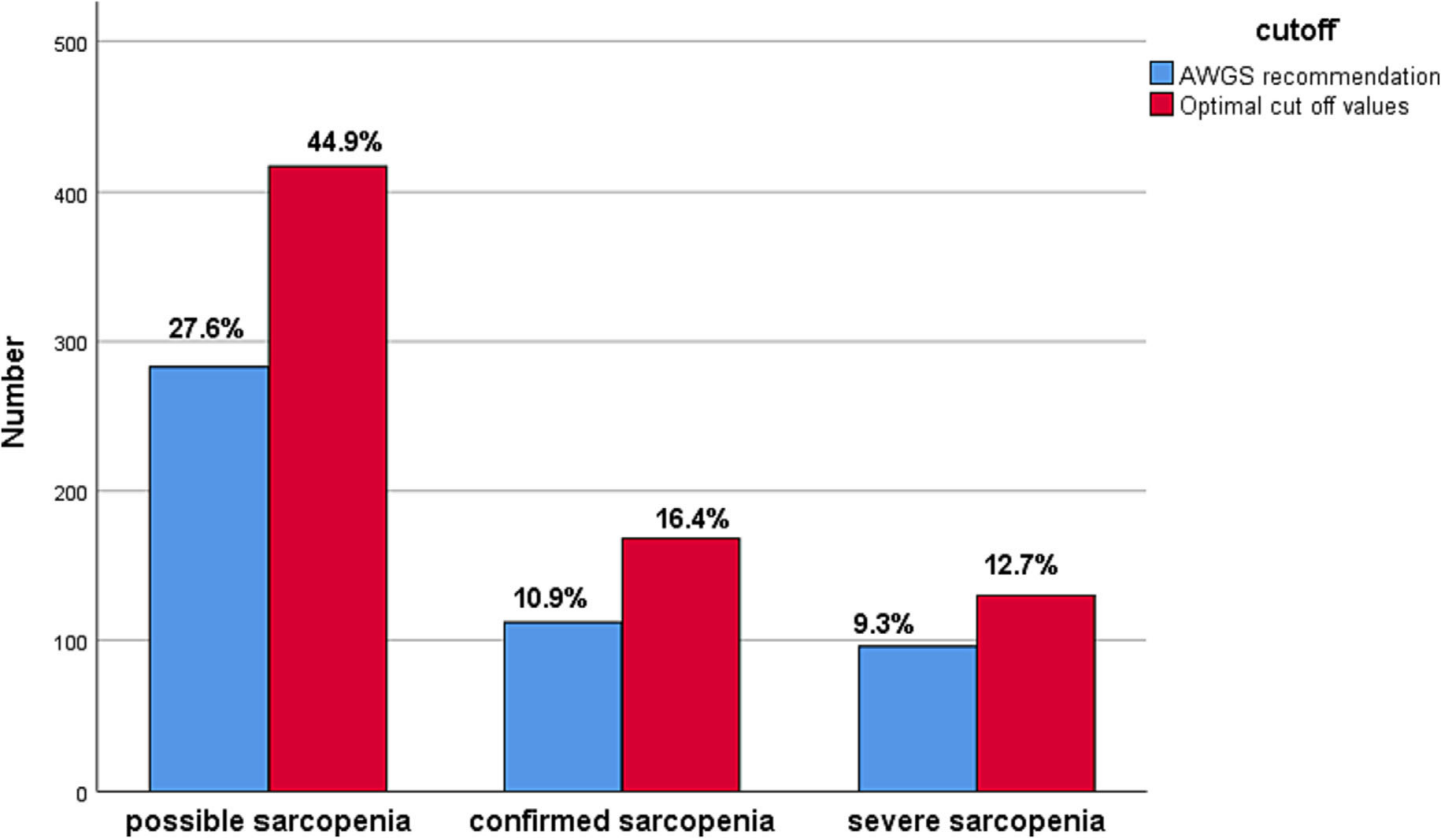
The change in the prevalence of sarcopenia using the optimal cut‐off values of five‐time chair stand test. AWGS, Asian Working Group for Sarcopenia.

*Table* *4* and *Figure* *2* show that when using the 5CST cut‐off value recommended by the AWGS, the prevalence of possible, confirmed and severe sarcopenia was 27.6%, 10.9% and 9.3%, respectively. Using the redefined cut‐off value, the prevalence of possible sarcopenia, confirmed sarcopenia and severe sarcopenia was increased to 44.9%, 16.4% and 12.7%, respectively; the difference in prevalence between the two cut‐off values was statistically significant (*P* < 0.001).

**Table 4:** Changes in the prevalence of sarcopenia using optimal cut‐off values of five‐time chair stand test

	AWGS recommendation		Optimal cut‐off values		*P* value
	Number	Prevalence (%)	Number	Prevalence (%)	
Possible sarcopenia (5CST)	283	27.6	461	44.9	<0.001
Confirmed sarcopenia (5CST + SMI)	112	10.9	168	16.4	<0.001
Severe sarcopenia (5CST + GS + SMI)	96	9.3	130	12.7	<0.001

Abbreviations: 5CST, five‐time chair stand test; AWGS, Asian Working Group for Sarcopenia; GS, gait speed; SMI, skeletal muscle index.

### Accuracy of five‐time chair stand test in assessing sarcopenia

**Figure 3:**
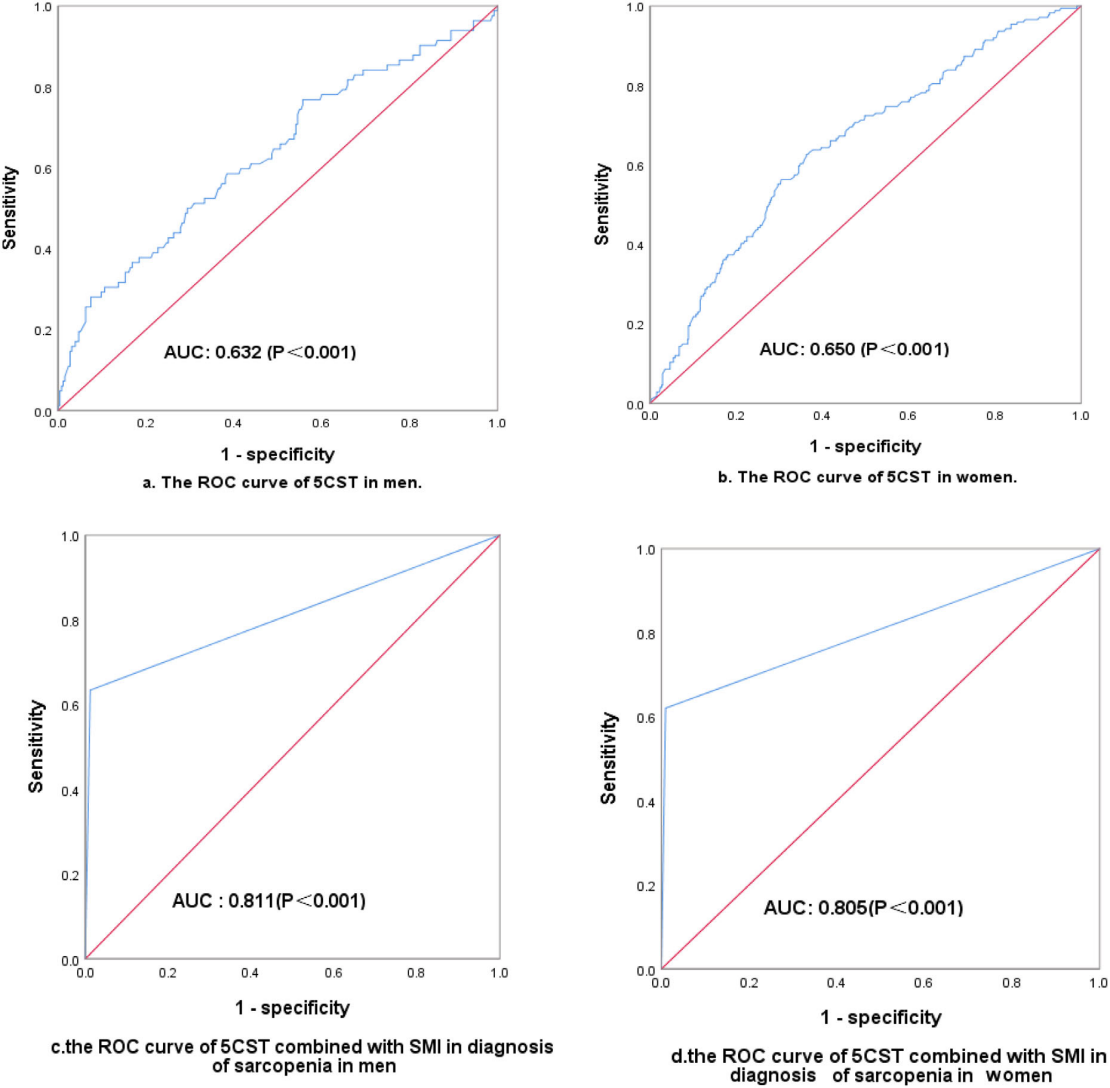
The receiver operating characteristic (ROC) curve of five‐time chair stand test (5CST) in assessing sarcopenia. Panels (A) and (B) show the ROC curve of 5CST in assessing sarcopenia in men and women, respectively. Panels (C) and (D) show the ROC curve of 5CST combined with skeletal muscle index (SMI) in the diagnosis of sarcopenia in men and women, respectively. The Asian Working Group for Sarcopenia criteria were used as the ‘gold standard’. AUC, area under the curve.

*Figure* *3* shows that 5CST had an area under the curve (AUC) of 0.632 (95% confidence interval [CI], 0.560–0.704) in men and 0.650 (95% CI, 0.604–0.694) in women (both *P* < 0.001). Combined with SMI, the AUCs were 0.811 (95% CI, 0.745–0.877) in men and 0.805 (95% CI, 0.760–0.851) in women (both *P* < 0.001). *Table* *5* shows that when 5CST alone was used for the assessment of sarcopenia, the sensitivity was 62.5%, the specificity was 61.0% and the Kappa value of the agreement test between 5CST and the gold standard was 0.185 (*P* < 0.001). Combined with SMI, the sensitivity was 62.5%, the specificity was 97.0% and the Kappa value was 0.700 (*P* < 0.001).

**Table 5:** Agreement between five‐time chair stand test and the Asian Working Group for Sarcopenia criteria in assessing sarcopenia

	AWGS criteria			Sensitivity (%)	Specificity (%)	Kappa value	*P* value
5CST		+	−				
	+	160	301	62.5	61.0	0.185	<0.001
	−	96	470				
5CST + SMI	+	160	8	62.5	97.0	0.700	<0.001
	−	96	763				
	+	107	23	71.8	97.4	0.731	<0.001
5CST + GS + SMI	−	42	855				

Abbreviations: 5CST, five‐time chair stand test; AWGS, Asian Working Group for Sarcopenia; GS, gait speed; SMI, skeletal muscle index.

## Discussion

To the best of our knowledge, our study is the first to determine the optimal cut‐off value of the 5CST to assess sarcopenia in Chinese community‐dwelling older adults. The key findings of our study are as follows: (1) 5CST was not significantly different between the genders in the total study population; the optimal cut‐off value of 5CST in assessing sarcopenia in the total population was 10.9 s. Stratified by age and gender, 5CST is different in different age groups and genders; we gave the optimal cut‐off value of 5CST in different age groups and genders. (2) The optimal cut‐off values of 5CST determined in our study can improve the detection rate of sarcopenia among community‐dwelling older adults.

In our study, the optimal cut‐off value of 5CST in assessing sarcopenia in the total population was 10.9 s. Stratified by age, for the older adults aged 60–69 and 70–79 years, 5CST was significantly different between the genders, but there was no difference between the genders in the older adults aged over 80 years. Our study gave the optimal cut‐off value of 5CST in different age groups and genders. For the older adults aged 60–69 years, the optimal cut‐off values of 5CST for the assessment of sarcopenia were 9.2 s in men and 10.8 s in women; for the older adults aged 70–79 years, cut‐off values were 10.2 s in men and 10.9 s in women; and for the older adults over 80 years, cut‐off values were 14.0 s in men and 11.5 s in women (all *P* < 0.001). In terms of the results of our study (*Table* *2*), 5CST is significantly different between men and women in older adults before the age of 80 years, and it is necessary to give specific cut‐off values by gender stratification. For older people over 80 years with poor body and muscle condition, the gender difference of 5CST will be weak or even no difference,^22^ and the assessment of 5CST may not need to be stratified by gender.

The optimal cut‐off value determined by our study can improve the detection rate of sarcopenia. The AWGS and European Working Group on Sarcopenia in Older People (EWGSOP) consensus set 12 and 15 s, respectively, as thresholds for low muscle strength or low physical performance to evaluate for sarcopenia preliminarily.^1^, ^2^ When using the 12 s as a cut‐off recommended by the AWGS diagnosis criteria, the prevalence of possible sarcopenia, confirmed sarcopenia and severe sarcopenia was 27.6%, 10.9% and 9.3%, respectively. Using the redefined optimal cut‐off value, the prevalence of possible sarcopenia, confirmed sarcopenia and severe sarcopenia was increased to 44.9%, 16.4% and 12.7%, respectively. Our results show that the newly determined cut‐off value can significantly improve the detection rate of sarcopenia. A study from Singapore is consistent with our findings. The study compared the prevalence of sarcopenia in older adults with different cut‐off values in the 5CST. The prevalence of possible sarcopenia was 6.5% when the cut‐off value was 15 s, and it increased to 15.5% when the redefined cut‐off value was 12.5 s.^23^ Determining the 5CST cut‐off value appropriate for the domestic population can promote the detection of sarcopenia in the community.

The cut‐off value of the 5CST assessment for older adults with sarcopenia varies between countries. The study of Gao et al. showed that the normal reference value of the 5CST of healthy Chinese adults was 10.23–14.06 s in men and 11.36–14.89 s in women.^24^ In our study, the cut‐off value of 5CST in assessing for sarcopenia was 10.9 s in the total population. A cross‐sectional study from South Korea showed that the cut‐off value of 5CST in assessing sarcopenia was 12.8 s in Korean older adults.^18^ The cut‐off value defined in their study was higher than the 10.9s determined in our study, which may be related to the fact that the older adults included in their study were all over 70 years old, but my study population was over 60 years old. A meta‐analysis showed that among Japanese older adults, the reference value for the 5CST was 8.50 s.^25^ Pinheiro et al.'s study suggested that the cut‐off value of 5CST for assessing sarcopenia was 13.0 s among older women in the Brazilian community.^26^ These studies all show that the performance of the 5CST among older adults in different countries is significantly different; it may also be due to the differences in physical fitness of the older adults in different countries,^27^ which leads to the different cut‐off values of the 5CST in screening for sarcopenia. It is important and necessary to redefine the optimal cut‐off value of the 5CST for screening sarcopenia among domestic older adults.

The performance of 5CST is limited when 5CST is used independently to assess sarcopenia. The EWGSOP recommended the 5CST and HS as interchangeable preliminary indicators for assessing sarcopenia; when either HS or 5CST is below the threshold, it is defined as possible sarcopenia, and when one of the two is below the threshold combined with a reduced SMI, it is defined as confirmed sarcopenia.^28^ In our study, with AWGS criteria as the gold standard, the AUC of 5CST in assessing sarcopenia is 0.632 in men and 0.650 in women; accuracy is fair. Combined with SMI, it is 0.811 in men and 0.805 in women (*P* < 0.001); accuracy is good. The important reason for the poor performance of 5CST in the assessment of sarcopenia may be that 5CST is affected by factors such as chair height, posture, balance, reaction time and cognitive and psychological status of older adults.^29^ It is important to standardize test procedures (such as chair height regulation and uniform posture) and avoid the influence of confounding factors when using 5CST to assess sarcopenia.

Our study has some strengths and limitations. This study is almost the first to determine the optimal cut‐off value of the 5CST to assess sarcopenia in Chinese community‐dwelling older adults. The newly determined cut‐off value can significantly improve the detection rate of sarcopenia in community‐dwelling older adults, which has far‐reaching significance for the early screening of sarcopenia. Our study suggests that it may be necessary to stratify 5CST by age and gender, which has not been given sufficient attention in previous studies. However, our study has the following limitations: First, our study only included the community of older adults in Hunan Province, and more studies are needed to confirm whether the redefined cut‐off value is suitable for all older adults in China. Second, our study showed that there was no significant difference between genders, and gave the cut‐off value of 5CST in the total population. However, due to the participants included in our study, the number of women was significantly higher than that of men, so the cut‐off value for the total population (10.9 s) should be used with caution. Lastly, we used BIA to assess muscle mass. BIA is sensitive to hydration status, temperature, measurement time, body symmetry and position, which may lead to less accurate muscle mass measurements than those obtained by computed tomography (CT) and magnetic resonance imaging (MRI).^30^, ^31^

## Conclusions

In this study, we determined the optimal cut‐off value of the 5CST for assessing older adults with sarcopenia in the Chinese community. The cut‐off value can significantly improve the detection rate of sarcopenia among community‐dwelling older adults, which will help community workers detect more people with sarcopenia and benefit early screening and management of sarcopenia in practice.

## Conflict of interest statement

The authors declare no conflicts of interest.

## Funding

Science and Technology Department of Hunan provincial (grant no. 2018SK21312).
